# Fluorescence Assay for the Determination of d-Panthenol Based on Novel Ring-Fused 2-Pyridone Derivative

**DOI:** 10.3390/ijms21218386

**Published:** 2020-11-09

**Authors:** Wiktor Kasprzyk, Tomasz Świergosz, Filip Koper

**Affiliations:** 1 Department of Biotechnology and Physical Chemistry, Faculty of Chemical Engineering and Technology, Cracow University of Technology, Warszawska 24, 31-155 Kraków, Poland; filipkoper1@gmail.com; 2 Department of Analytical Chemistry, Faculty of Chemical Engineering and Technology, Cracow University of Technology, Warszawska 24, 31-155 Kraków, Poland; tomasz.swiergosz@pk.edu.pl

**Keywords:** d-panthenol, citric acid, fluorescence, derivatization, ring-fused 2-pyridones

## Abstract

Herein, a novel fluorescent method for the determination of d-panthenol (DP) level in solutions with no separate hydrolysis step has been revealed based on the utilization of citric acid (CA) as a derivatizing agent. Consequently, the essential parameters of the derivatization process were established, resulting in the development of sensitive, repeatable, and accurate determination of panthenol. The method was approved, and its usefulness in characterizing the concentration of DP in pharmaceutical formulations and selectivity in the determination of DP were validated. The chemical structure of the new fluorophore formulating in the reaction in DP with CA, i.e., 6-oxo-3,4-dihydro-2*H*,6*H*-pyrido[2,1-*b*][1,3]oxazine-8-carboxylic acid (ODPC), was elucidated using detailed NMR experiments: one-dimensional (^1^H, ^13^C) as well as two-dimensional NMR spectra (^1^H-^1^H COSY, ^1^H-^13^C HSQC, ^1^H-^13^C HMBC, ^1^H-^15^N HSQC, ^1^H-^15^N HMBC).

## 1. Introduction

d-panthenol (DP) (IUPAC name: (2R)-2,4-dihydroxy-*N*-(3-hydroxypropyl)-3,3-dimethylbutanamide) is an alcohol analog of vitamin B5 (pantothenic acid). Due to its enhanced stability, panthenol is mainly used as an ingredient in many pharmaceutical and cosmetical formulations. It has moderate irritating properties and is non-allergenic and not mutagenic [1,2,3]. After application of d-panthenol to the skin, it is readily absorbed and transformed into pantothenic acid, promoting new cell growth, binding water, and acting as a moisturizer. Vitamin B5 is an important component of Coenzyme A, known for its vital role in the metabolism of carbohydrates, fats, and nitrogen compounds. Lack of pantothenic acid causes many types of deficiency diseases, skin irritation, dermatitis, hair depigmentation, and growth inhibition [4]. DP is applied externally to increase the epithelialization of burns and scratch wounds and ulcers [5,6,7]. It is associated with the fact that pantothenic acid is essential for maintaining the proliferation and differentiation of keratinocytes as it regulates the epidermal barrier function by proliferation and differentiation of keratinocytes directly or indirectly through the synthesis of KGF (Keratinocyte Growth Factor) and type IV collagen [6].

DP as an aliphatic alcohol free of conjugated bonds and chromophores does not exhibit absorption in either the near-ultraviolet (300–400 nm) or the visible part of the electromagnetic spectrum. Nevertheless, direct spectrometric methods for the determination of DP after chromatographic separation have been reported [8,9,10,11]; however, these suffer from a lower sensitivity [8]. Other direct DP assays characterized in the literature include NMR [12], electrochemical [1], GC-FID [4,13], and LC-MS [8] detection of DP concentration. In order to improve the sensitivity of DP assays, some derivatization methods have been introduced. In most cases, these methods consist of hydrolysis of DP for the production of β-alanol (3-amino-1-propanol), a primary amine susceptible to the reaction of nitrobenzoxadiazole (NBD) and ninhydrin for the formulation of colored or fluorescent derivatives [12,13,14,15,16,17,18,19]. Thus, using fluorescence DP assays, it is possible to easily achieve LOD (Limit of Detection) as low as 10^−8^ g/mL [12]. However, the above methods use HPLC separation or tedious DP extraction steps before the actual reaction with a derivatizing agent in order to reach an appropriate level of selectivity. The latter fact is associated with the ability of NBD and ninhydrine to react with all α-amine-containing components of a studied sample and not only 3-amino-1-propanol [20,21]. Therefore, the requirement for a novel, simple, inexpensive, and sensitive protocol of DP determination in various complex formulations seems indisputable.

Citric acid (CA) is one of the most prominent carboxylic acids found in living organisms. CA is a crucial intermediate in the Krebs cycle occurring in the metabolism of all aerobic organisms [22]. Recently, CA has been investigated in terms of possible use as an inexpensive carbon source for bottom-up, hydrothermal synthesis of carbon dots [23,24,25] which resulted also in the development of a series of novel highly photoluminescent organic fluorophores, i.e., derivatives of ring-fused 2-pyridones [26,27,28,29,30,31,32,33]. The latter fluorophores were found to be a product of the condensation of CA and specific β-amines [29]. Taking into consideration the fact that DP moiety hydrolyzes to produce pantolactone (PAN) and 3-amino-1-propanol (3A1P) upon heating [34], it is reported that the latter is able to react with CA to produce a fluorescent derivative of 2-pyridone, simultaneously. Therefore, it was shown that by a careful choice of the derivatization conditions, the fluorescent signal of the reaction solution becomes proportional to the quantity of DP in the investigated samples. Thus, in this research, a novel spectrofluorimetric method for DP determination in various pharmaceutical and cosmetic formulations without a separate hydrolysis step of panthenol was introduced.

## 2. Results and Discussion

Considering that the concept of implementing CA as a derivatizing agent for DPs in the preparation of DP assay has not been described yet, it has been found necessary to elucidate the principles of the proposed procedure. The first paragraphs, consequently, discuss the chemical origin of the fluorescent signal that appears after the CA and DP thermal reaction. As a result, to determine the most optimal reaction conditions for the efficient synthesis of fluorescent molecules, three reaction parameters were differentiated, i.e., molar ratio CA:DP, temperature, and reaction time.

**Figure 1:**
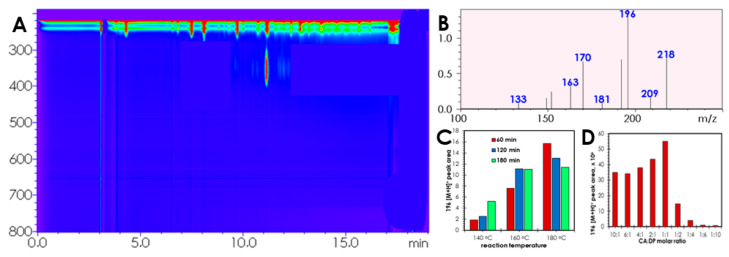
LC-DAD chromatogram of CA:DP reaction mixture (1:1 mol/mol, 160 °C, 180 min) (**A**), mass spectra of fractions with retention times ~11.8 min (**B**,**C**) determination of the most optimal reaction conditions for the efficient synthesis of the fluorescent molecules (**D**), determination of the ratio of CA:DP reagents for the most efficient fluorophore synthesis.

Therefore, several reactions differing in the molar ratio CA:DP were conducted, and the resulting reaction mixtures were dissolved in methanol and analyzed with LC-DAD-MS. On the LC-DAD chromatogram of the methanolic solutions of reaction mixtures, one main peak and several minor fractions absorbing around 350 nm were observed, indicating that the fluorophore was formed as a major component during the reaction (Figure 1A). The most prominent fraction (retention time 11.8 min) was, therefore, investigated to reveal the nature of the proposed derivatization process. The above-mentioned fraction was found to have a molecular weight of 195 Da (*m/z* 196 [M + H]^+^) (Figure 1B). Moreover, comparing *m/z* 196 [M + H]^+^ peak areas of chromatograms of samples prepared at various molar ratios of CA:DP, it was found that for the efficient synthesis of the fluorophore, the molar ratio of reagents 1:1 was the most suitable (Figure 1D). According to subsequent investigations, an increase in temperature and reaction time leads to a notable improvement in reaction efficiency; on the other hand, an increase in reaction time, especially when combined with high reaction temperature, results in a significant decrease in the area of the LC-DAD fluorophore peak (Figure 1C). The above fact is probably associated with the thermal decomposition of the product and/or polymerization as well as carbonization of the precursors leading to the formation of less PL emitting moieties, including carbon and graphene dots [35]. Ultimately, it was possible to synthesize and separate fluorophore in the proper amount and purity, thereby allowing for comprehensive characterization studies of the chemical structure (Figure S1).

Simultaneously, CA was reacted with 3-amino-1-propanol and separately with pantolactone (thermal degradation products of panthenol) [19,34] and resulting reaction mixtures were dissolved in methanol and analyzed with LC-DAD-MS. The above analyses were aimed at identifying the part of the DP molecule that is capable of reacting with CA to form a compound that absorbs around 350 nm. This resulted in the conclusion that only in the reaction with 3-amino-1-propanol CA transforms into a molecule with absorption capacity at ~350 nm (Figure S2). Furthermore, the mass analyses revealed that the same compound with a molecular weight of 195 Da is formed following the reaction of CA with DP and CA with 3-amino-1-propanol (Figure S3). All the above indicates that during the derivatization of DP with CA, the DP molecule undergoes thermal hydrolysis, releasing 3-amino-1-propanol molecules capable of formation of the fluorescent compound in condensation with CA. Given the above observations and our previous experience in the synthesis of fluorescent compounds from citric acid, it was assumed that fluorophore contains ring-fused 2-pyridone derivative (6-oxo-3,4-dihydro-2*H*,6*H*-pyrido[2,1-*b*][1,3]oxazine-8-carboxylic acid) (ODPC), similar to those reported earlier in the literature [29].

**Figure 2:**
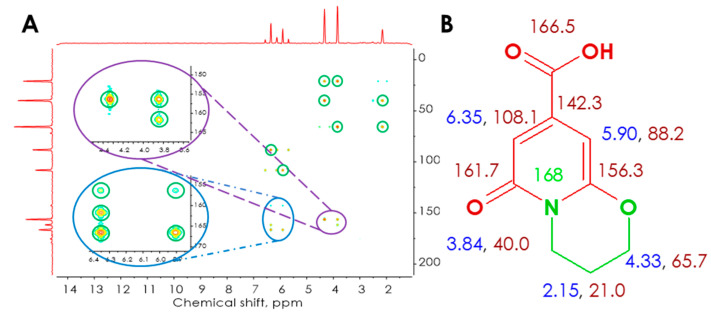
^1^H–^13^C HMBC spectra of ODPC with appropriate extensions—significant correlation signals marked with green circles (**A**). Chemical structure of ODPC with relevant NMR assignments (**B**).

In accordance, the proposed chemical structure of the blue-emitting moiety was undeniably confirmed in the form of in-depth NMR studies of pure compound isolated from the post-reaction mixture. In the ^1^H NMR spectrum, two doublets (δ 6.35 and 5.90 ppm), two triplets (δ 4.33 and 3.84 ppm), and one quintet (δ 2.15 ppm) with integrals ratio 1:1:2:2:2 were found (Figure S4). Following the COrrelation SpectroscopY (COSY) ^1^H–^1^H experiment (Figure S5), four signals of correlated protons were shown, i.e., 6.35–5.90, 3.84–4.33, 3.84–2.15, 4.33–2.15 ppm. The final two correlations indicate couplings of geminal methylene protons of the six-membered aliphatic ring, while signals at δ 6.35–5.90 and 3.84–4.33 ppm were ascribed to long-distance correlations of methylene protons neighboring heteroatoms of the same ring and methine protons of the pyridine ring, respectively. For ^13^C NMR, eight carbons lying in different chemical environments were observed (carbonyl—δ 166.5, 161.7 ppm; heterocyclic—δ 88.2, 108.1, 142.8, 156.3 ppm; aliphatic—21.0, 65.7) (Figure S6). The signal originating from one of the aliphatic carbons of the fluorophore moiety was not observed in ^13^C NMR spectra due to overlapping solvent signals. Fortunately, the above signal could be easily ascribed using the HSQC ^1^H–^13^C experiment, where the coupling of carbon with chemical shift δ 40.24 ppm with methylene protons δ 3.85 ppm was detected. The HSQC ^1^H–^13^C spectrum of the examined fluorophore further confirmed similarities in its chemical structure to previously reported 2-pyridone-based fluorophores, where signals originating from direct couplings of protons with appropriate carbons of the heterocyclic ring could be recognized (6.35–108.1, 5.91–88.13) [31]. The remaining two signals were ascribed to couplings between protons and carbons of methylene groups neighboring heteroatoms in a six-membered aliphatic ring (4.33–65.72, 2.14–20.98) (Figure S7). The ^1^H-^15^N HSQC and HMBC spectra (Figures S8 and S9) revealed that the fluorophore moiety is constituted by one nitrogen atom without a directly attached proton. Nevertheless, the nitrogen shows long-distance correlations characteristic of the pyridinic nitrogen of ring-fused 2-pyridone derivatives (6.35–168, 5.90–168, 3.84–168, 2.15–168 ppm). The multiple long-distance correlations between protons and carbons of the studied fluorophore (Table S2) were observed in the HMBC ^1^H–^13^C spectra (Figure 2A) and allowed for unambiguous confirmation of its chemical structure, which is constituted by 6-oxo-3,4-dihydro-2*H*,6*H*-pyrido[2,1-*b*][1,3]oxazine-8-carboxylic acid (ODPC) (Figure 2B).

**Figure 3:**
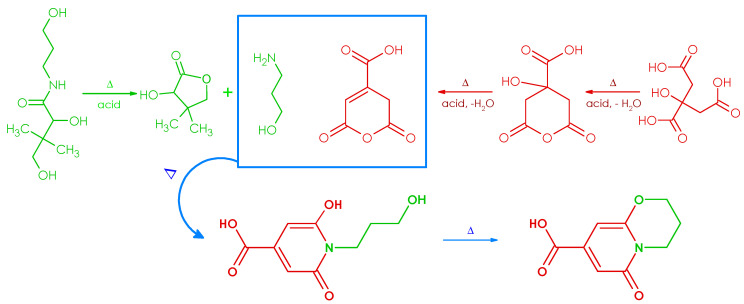
The possible mechanism of ODPC formulation in the course of thermal condensation of CA with DP.

Thus, the probable mechanism of its formulation, in the course of thermal condensation of CA and DP, requires the formation of six-membered anhydride originating from dehydration of CA, thermal hydrolysis of amide linkage of DP resulting in the release of a 3-amino-1-propanol moiety, and inter- and intramolecular condensation of CA anhydride with 3-amino-1-propanol (Figure 3). The above investigations concerning the elucidation of the chemical structure of fluorescent dye may also be relevant to shed light on the molecular origin of the high PL quantum yield of many carbon dots prepared from citric acid and aminoalcohol derivatives reported so far [36,37,38].

**Figure 4:**
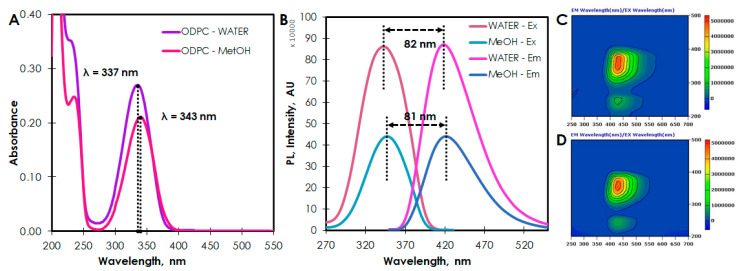
UV/VIS absorption spectrum of the aqueous and methanolic solution of ODPC (**A**), PL excitation and emission spectra of aqueous and methanolic solution of ODPC (**B**), and 3D excitation and emission spectra of aqueous (**C**) and methanolic (**D**) solution of ODPC.

Concerning the spectroscopic properties of ODPC, it was found that the aqueous solution of the above-mentioned fluorophore exhibits strong absorption bands of around 230 and 337 nm (343 nm for methanol ODPC solution) probably originating from the π → π* transition of sp^2^ carbons of the heterocyclic 2-pyridone ring and to the n → π* transition of C=O bonding (Figure 4A). ODPC exhibits excitation-independent PL spectra with a maximum emission wavelength at 419 nm (421 nm for the methanolic solution of ODPC) and a maximum of excitation wavelength at 342 nm (347 nm for the methanolic solution of ODPC) (Figure 4B–D). Broad and symmetrical PL emission spectra of ODPC display common tailing and Stokes shifts (82 nm for an aqueous and 81 nm for a methanolic solution, respectively) reported for other organic fluorophores [39,40,41]. The PL QY of solutions of ODPC determined using the comparative method was 0.53 for methanolic and 0.64 for aqueous solutions. Consequently, ODPC resembles ring-fused 2-pyridone derivatives obtained in the reaction of CA and α,β-diamines, β-aminoalcohols, and β-aminothiols in terms of optical properties [33].

**Figure 5:**
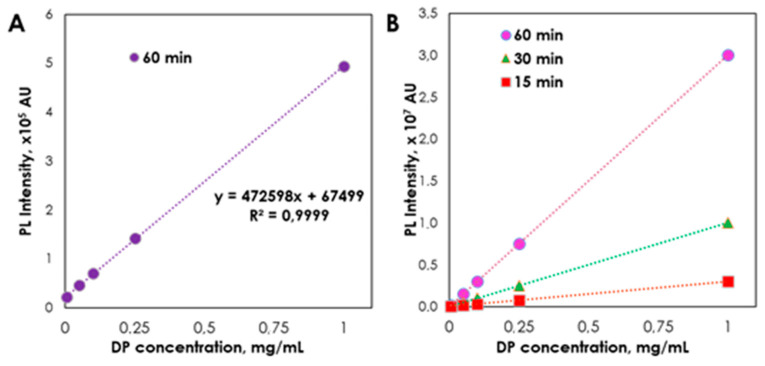
Linearity of the citric acid–panthenol derivatization process for solutions with different DP concentrations carried out for 60 min in sealed vials with needles in caps (**A**), at different reaction times (**B**).

Finally, in light of DP’s ability to form highly fluorescent ring-fused 2-pyridone derivatives under thermal condensation with CA, the development of a CA-based DP assay using CA as a derivatizing agent has become feasible. Nevertheless, some crucial parameters of the derivatization process had to be precisely defined before proceeding with actual plotting of the DP assay calibration curve (i.e., CA:DP molar ratio, type of vessel used, time, and temperature). Although Figure 1D shows that the highest efficiency of ODPC formation under non-aqueous conditions can be achieved with a molar ratio of 1:1 CA:DP, the design of the functional DP assay requires to a large extent a derivatizing agent (despite the decrease in reaction efficiency). The latter fact is associated with the obligation that the developed protocol should be insensitive to possible CA-based additives in investigated samples, and the DP concentration vs. PL emission plot should be linear over a wide range of analyte concentrations. Therefore, throughout all experiments for the DP assay development, an excess of CA as a derivatizing agent was used. For the derivatization time and temperature, it was established that the process had to be conducted for 60 min at 160 °C to ensure high sensitivity of the protocol within an acceptable period of time (Figure 5B). It was, furthermore, concluded that it is critical to carry out the derivatization of DPs in vessels with controlled release (Figure S10) to avoid rapid evaporation of aqueous solutions causing significant inhibition of ODPC formation and, in extreme cases, leading to a complete failure to apply the DP assay. Concluding all the above, the final conditions of derivatization that are relevant to the DP assay under consideration comprise the heating of the reagents for 60 min at 160 °C in sealed vials with needles in caps and application of 150 mg/mL of CA (1.5 mL) and 1.5 mL of a sample. Thereafter, DP aqueous solutions were subject to derivatization with CA and resulting reaction mixtures were dissolved in methanol and their PL emission spectra were analyzed (λ_ex_ = 347 nm, λ_em_ = 400–600 nm), enabling plotting of the DP calibration curve (Figure 5A). The latter indicates the high accuracy and reproducibility of the protocol (R^2^ = 0.999) within a broad DP concentration range, indicating good linearity. In order to evaluate the applicability of the developed DP assay, three different DP-containing pharmaceutical formulations supplied in the form of creams, lotions, and nasal spray applicators were analyzed. Hence, the resulting DP levels in the above products correlated well with the amounts of DP declared by the manufacturers and DP concentrations determined by the LC-ESI-MS method (Table 1) from the calibration curve (Figure S11).

Moreover, the precision of the proposed methods was determined in the range of concentrations presenting linearity, performing three measurements each on different days of the week. The target concentrations corresponded to the average values in each of the concentration ranges. Table 1 shows RSD concentrations and accuracy of 0.005, 0.05, and 0.1 mg/mL solutions determined according to the proposed procedure.

Three replications for the determination of panthenol were performed. Moreover, in order to evaluate the suitability of the proposed methods, a standard method of adding panthenol to the previously analyzed pharmaceutical products was used. The recovery of each product was calculated by comparing the concentration obtained from (spiked) mixtures with the concentration of pharmaceutical products. Table 2 presents the results of the analyses of commercial formulations with recovery (standard addition method) of panthenol. The comparison of the results obtained with the proposed method available in the literature [42,43] shows that the accuracy of the results is acceptable.

These procedures are suitable for routine quality control of the compounds to be tested with minimal interference, due to the high-intensity fluorescent bands obtained and the absence of derivatizing reagent background. The proposed and reference methods have been used for the determination of panthenol-containing tested pharmaceutical products. The average values (±S.D.) of the amounts determined varied slightly, indicating similar precision and accuracy. The results obtained indicate that the formation of fluorophore between citric acid and panthenol is favored and the reaction between the two chemical compounds can be used for spectrofluorometric analyses. Therefore, it became clear that CA could serve as an inexpensive DP derivatization agent which enables sensitive fluorescent determination of DP concentration in a variety of samples.

## 3. Materials and Methods

### 3.1. Materials

Citric acid (CA) (Sigma-Aldrich, Saint Louis, MO, USA), d-Panthenol (DP) (Fluorochem, Derbyshire, UK), 3-amino-1-propanol (3A1P) (Sigma-Aldrich), pantolactone (PAN) (Fluorochem), Coumarin 1 (Sigma-Aldrich), methanol MS grade (Avantor, Gliwice, Poland), acetone gradient grade (Avantor), ethanol (Avantor), dimethyl sulfoxide-d6 (Sigma-Aldrich). All chemicals and solvents were of analytical grade and used as received. Water used throughout the experiments with a resistivity of 18.0 mΩ cm^−1^ at 295 K was deionized through a Purix water purification system.

### 3.2. Methods

#### 3.2.1. Synthesis, Separation, and Structure Elucidation of the Fluorophore

For the synthesis of the fluorophore to obtain the most favorable reaction conditions to elucidate its chemical structure, DP was reacted with different amounts of CA in glass vials at 160 °C for 3 h (Table S1A). Thereafter, each reaction mixture was dissolved in 7 mL of demineralized water using an ultrasonic bath. The prepared solutions of the post-reaction mixtures were evaluated by LC-ESI-MS analysis and the *m/z* 196 [M + H]^+^ chromatogram peak areas of fractions with retention time 11.8 min were compared. Then, equimolar mixtures of CA (242 mg) and DP (258 mg) were heated at various temperatures (140, 160, and 180 °C) and for different periods of time (1, 2, and 3 h). Afterwards, each reaction mixture was dissolved in 7 mL of methanol and subjected to LC-ESI-MS analysis. Furthermore, to establish which part of DP chemical structure is responsible for formulation of fluorophore in reaction with CA, reactions of CA with DP thermal degradation products were conducted. Thus, CA (300 mg) was reacted with 3-amino-1-propanol (117 mg) (Table S1B) and pantolactone (203 mg) (Table S1C) in separate vials for 3 h at 160 °C and then the reaction mixtures were dissolved in methanol and analyzed using LC-MS. For isolation of pure fluorophore, 1451 mg of CA and 1549 mg of DP (molar ratio 1:1) were weighed into a 7 mL vial and reacted for 1 h at 180 °C in a heating block at 100 rpm. The sample after cooling was dissolved in 14 mL of methanol and then subjected to the next stage—separation by preparative liquid chromatography. The appropriate fluorescent fraction was isolated from the reaction mixture, freeze-dried, and its purity was confirmed using LC-DAD-ESI-MS analysis.

To characterize the elemental composition of the fluorophore, high-resolution mass spectrum was acquired using the MALDISynapt G2-S HDMS (Waters Corporation, Milford, MA, USA), coupled to a Waters TQD mass spectrometer (electrospray ionization mode ESI–tandem quadrupole). A constant flow of 0.3 mL/min and two mobile phases were used: A-phase: 0.1% formic acid and B-phase: methanol. For chemical structure elucidation of the fluorophore ^1^H and ^13^C, COSY ^1^H-^1^H, ^13^C-^1^H HSQC and HMBC, ^15^N-^1^H HSQC, and HMBC experiments were performed. NMR analyses were recorded on Avance III HD 400 MHz (Bruker) in dimethyl sulfoxide-d6 as a solvent.

#### 3.2.2. Conditions of Chromatographic Separation

For analytical low-resolution LC-ESI-MS/MS analyses, LCMS-8030 (Shimadzu, Kyoto, Japan) mass spectrometric system coupled to an LC-20ADXR pump utilizing the LC gradient was used. The LC analyses were carried out on a 100 mm × 4.6 mm × 5.0 μm Kinetex C18 chromatographic column (Phenomenex, Torrance, CA, USA). Kinetex C18 chromatographic column was preceded by a guard column of the same material (Phenomenex, Torrance, CA, USA). The positive ion chromatograms and mass spectra were recorded by LabSolutions software version 5.91 (Shimadzu). The injection volume was 5 µL, and the flow rate was 0.5 mL/min. The column was thermostated at 40 °C. The separation of the analytes was performed with binary gradient elution. The mobile phases were: A—2% formic acid in the demineralized water, and B—pure methanol. The gradient profile was (t [min], % B), (0, 10), (13, 40), (15, 90), (17, 90).

A flash chromatography system was employed to isolate fluorescent fractions from reaction mixture solutions. Preparative HPLC system with LC-20AP pumps, UV–VIS SPD-20AV detector, and LabSolutions 5.51 operating software (Shimadzu, Japan) equipped with Kinetex 5 µm C18 100A, AXIA Packed HPLC Column 250 × 21.2 mm preceded by a guard column of the same material was applied. Gradient elution was utilized consisting of phase A, which was 2% formic acid, and phase B, methanol. The mobile phase flow was 20 mL/min, and the injection volume was 20 mL. The column was thermostated at 40 °C. The gradient profile was (t (min), % B), (0, 10), (33, 40), (38, 90).

#### 3.2.3. Optical Properties Characterization

UV-VIS absorption spectra of methanolic and aqueous solutions of the purified fluorophore as well as the ethanolic solution of coumarin 1 were analyzed. Samples were prepared by dissolving the previously freeze-dried compound in the above-mentioned solvents. The samples with a concentration of 0.002 mg/mL were subjected to recording spectra in range 200–600 nm. For the analyses, a UV-2600i spectrophotometer from Shimadzu and quartz cuvettes with an optical path of 1 cm were used.

Fluorescence emission and excitation spectra of aqueous solutions of reaction mixtures, pure fluorophore, an ethanolic solution of coumarin 1 were acquired using RF-6000 spectrofluorometer (Shimadzu, Kyoto, Japan), both emission and excitation slits were set at 3 nm, and an L-42 optical filter was used (340–420 nm). Fluorescence 3D spectra of the fluorophore were measured using the same equipment, excitation and emission slits were set at 3 nm, excitation range 250–500 nm, emission range 250–800 nm.

Fluorescence quantum yield (QY) determinations were conducted using a comparative method using coumarin 1 (fluorescence max 447 nm, useful excitation range 320–420 (max 376) nm, absolute QY = 0.73 in ethanol [44])) as standard. All solutions were prepared in the absorption range 0.05–0.1 (355 nm) to eliminate concentration quenching effects, and an L-42 optical filter was used (340–420 nm). The samples were excited at 355 nm, and the fluorescence spectra were acquired between 380 and 700 nm. The QY was calculated as follows:

$$\Phi =\Phi_{st}\frac{n^{2}\left(1-10^{-A_{st}}\right)\int I_{em}dv}{n_{st}^{2}\left(1-10^{-A}\right)\int I_{em}^{st}dv}$$

where:

Φ and $\Phi_{st}—$the quantum yield of a sample and the standard;
A and $A_{st}—$absorption of a sample and the standard at 408 nm;
$\int I_{em}dv$ and $\int I_{em}^{st}dv—$integral of fluorescence intensity of a sample and the standard;
n and $n_{st}—$the refractive index of solvent used for a sample and the standard.

### 3.3. Determination of DP Concentration

#### 3.3.1. Preparation of Standard Solutions

Stock standard solutions were prepared fresh before use by dissolving the weighed solid standard in deionized water (DP: molar mass 205.25 g/mol and CA: molar mass 192.12 g/mol). They were further diluted with deionized water to obtain working solutions for derivatization. For DP (water solubility: 562.3 mg/mL; 20 °C), the concentrations of working solutions were as follows: 1; 0.25; 0.1; 0.05; 0.005 mg/mL. The concentration of CA working solution was 150 mg/mL (water solubility: 1630 mg/mL; 20 °C). The samples were stored in the dark, at low temperature (5 °C), for further processing. All solutions were filtered through a 0.20 µm nylon syringe filter (PTFE, Sartorius, Germany) and then degassed in an ultrasonic bath for 15 min.

#### 3.3.2. Derivatization of DP

For derivatization of DP, 1.5 mL of CA working solution was mixed with 1.5 mL of DP working solution in 7 mL glass vial reactors (in triplicate) equipped with a magnetic stirring bar. The vials were placed on a heating block, and the derivatization reaction occurred at 160 °C for 1 h. Finally, the reaction mixtures were dissolved in 7 mL of methanol, the PL emission spectra of the solutions were acquired, and the calibration curve was calculated. The wavelength for the PL excitation was 347 nm; PL emission range was set to 380–700 nm. All samples were degassed in an ultrasonic bath for 5 min before experiments and 15 min after the reaction to increase the dissolution process in methanol.

#### 3.3.3. Pharmaceutical Sample Preparation

The preparation of the pharmaceutical samples was considered crucial, with many interfering substances in the matrix. The first step was to separate the dissolved pharmaceutical in deionized water from insoluble additives. Therefore, optimization based on a simple, one-step sample preparation technique provides an especially useful tool for conducting reactions with pharmaceutical solutions. Consequently, 1 g of the formulation (50 mg DP declared) was transferred into a 500 mL volumetric flask and diluted with deionized water. The dissolution of the pharmaceutical was supported by an ultrasonic bath for 15 min. Then, the suspension was filtered using a syringe filter (PTFE, 0.20 μm); the resulting solution was diluted 10-fold (approx. 0.2 mg/mL) with deionized water and derivatized under the conditions specified above.

## 4. Conclusions

The chemical structure of the new fluorophore (ODPC) was elucidated using detailed NMR experiments, allowing us to suggest a possible mechanism for its formation involving the formation of six-membered anhydride originated from dehydration of CA, thermal hydrolysis of amide linkage of DP resulting in the release of a 3-amino-1-propanol moiety, and inter- and intramolecular condensation of CA anhydride with 3-amino-1-propanol. Furthermore, optical properties of ODPC resemble those ascribed to similar ring-fused 2-pyridone fluorophores reported earlier. A profound understanding of the chemical and optical properties of ODPC allowed for evolving a novel DP assay which involves the utilization of CA as a derivatization agent. Critical parameters of the derivatization procedure (reaction time, temperature, molar ratio, solvent, and fitting of reaction vessels) were established. The DP determination method demonstrates high accuracy and reproducibility (R^2^ = 0.999, accuracy 98.5–99.6%, precision (RDS%) 1.5–2.2) within a wide range of DP concentrations: 0.005–1 mg/mL. Furthermore, the established protocol proved to be of high accuracy in the determination of DP levels in pharmaceutical formulations (validated using LC-MS DP determination method). Thus, all the above argue in favor of the novelty of the presented work, while its complexity and diversity can become a starting point for various, more detailed studies and give rise to the development of novel fluorescence determination methods for a variety of important chemical species.

## Figures and Tables

**Table 1:** Validation of the method and results of the system applicability examination.

	Method			
Parameters	Fluorescence		LC-MS	
Regression equation	A = 472598x + 67499		A = 543834x + 63551	
Linear range (mg/mL)	0.005–1.000		0.005–1.000	
Limit of detection (mg/mL)	0.001		0.0005	
Limit of quantitation (mg/mL)	0.005		0.0075	
Correlation coefficients (*r*)	0.999		0.999	
Intraday precision and accuracy (mg/mL)	Accuracy, %	Precision, RSD %	Accuracy, %	Precision, RSD %
0.005	98.5	1.5	98.2	2.0
0.05	99.1	1.8	98.1	1.7
0.1	99.6	2.2	99.4	1.9

**Table 2:** Determination of panthenol in pharmaceutical formulations (*n* = 3).

Pharmaceutical Formulations	Fluorescence Method			LC-MS Method		
	Found (mg)	Equivalent Nominal Content ±S.D. (%)	Recovery (%)	Found (mg)	Equivalent Nominal Content ±S.D. (%)	Recovery (%)
nasal liquid	49.8	99.52 ± 1.10	99.85 ± 1.52	49.9	99.62 ± 1.11	99.02 ± 1.85
ointment	49.5	98.92 ± 1.52	99.56 ± 1.92	49.5	99.12 ± 2.52	99.33 ± 1.32
body lotion	49.2	98.10 ± 2.12	99.46 ± 1.82	49.4	98.80 ± 2.16	99.36 ± 1.12

## Supplementary Materials

The following are available online at https://www.mdpi.com/1422-0067/21/21/8386/s1. Table S1. Molar ratios for reactions of citric acid in glass vial reactors at 160 °C for 3 hours with DP (A), 3A1P (B), PAN (C). Figure S1. LC-DAD chromatogram of pure fluorophore separated from the reaction mixture (retention time: 11.8 min) (A). LC chromatogram of the pure fluorophore calculated from a maximum plot of 190–800 nm (retention time: 11.8 min) (B). Figure S2. LC-DAD chromatogram of CA:3A1P reaction mixture (1:1 mol/mol. 160 °C. 180 min) (A). and LC-DAD chromatogram of CA:PAN reaction mixture (1:1 mol/mol. 160 °C. 180 min) (B). Figure S3. The mass spectrum of the fraction with a retention time of 11.8 minutes formed in a reaction with a molar ratio from CA:31AP 1:1. Figure S4. ^1^H NMR of the fluorescent fraction. Figure S5. ^1^H-^1^H COSY NMR of the fluorescent fraction. Figure S6. ^13^C NMR of the fluorescent fraction. Figure S7. ^1^H-^13^C HSQC NMR of the fluorescent fraction. Figure S8. ^1^H-^15^N HSQC of the fluorescent fraction. Figure S9. ^1^H-^15^N HMBC of the fluorescent fraction. Table S2. Couplings between the atoms in ^1^H-^13^C HMBC NMR of the fluorescent fraction. Figure S10. The type of vessel used in the process of panthenol derivatization. Figure S11. LC-MS calibration curve for the determination of panthenol in pharmaceutical formulations (calculated from the area under the curves).
