# Supplementary material for: Fluorescence Assay for the Determination of d-Panthenol Based on Novel Ring-Fused 2-Pyridone Derivative
Source: Int J Mol Sci. 2020 Nov 9;21(21):8386. doi: 10.3390/ijms21218386 (PMC7664857; doi:10.3390/ijms21218386)
Supplement: Supplementary file 1 [file ijms-21-08386-s001.pdf]

S U P P L E M E N T A R Y   I N F O R M A T I O N

Fluorescence assay for the determination of D-panthenol based on novel ring-fused 2-pyridone derivative

Wiktor Kasprzyk[a*], Tomasz Świergosz[b], Filip Koper[a]

[1] Department of Biotechnology and Physical Chemistry, Faculty of Chemical Engineering and Technology, Cracow University of Technology, Warszawska 24, 31-155 Kraków, Poland; wiktor.kasprzyk@pk.edu.pl

[2] Department of Analytical Chemistry, Faculty of Chemical Engineering and Technology, Cracow University of Technology, Warszawska 24, 31-155 Kraków, Poland; tomasz.swiergosz@pk.edu.pl

* Correspondence: wiktor.kasprzyk@pk.edu.pl (W.K)

Table S1. Molar ratios for reactions of citric acid in glass vial reactors at 160 °C for 3 hours with DP (**A**), 3A1P (**B**), PAN (**C**).

A

Molar ratio CA:DP	Mass of CA [mg]	Mass of DP [mg]
10:1	451.76	48.24
6:1	424.47	75.53
4:1	394.66	105.34
2:1	325.98	174.02
1:1	241.81	258.19
1:2	159.47	340.53
1:4	94.86	405.14
1:6	67.51	432.49
1:10	42.82	457.18

B

Molar ratio CA:3A1P	Mass of CA [mg]	Mass of 3A1P [mg]
10:1	481.20	18.8
6:1	469.44	30.56
4:1	455.52	44.48
2:1	418.30	81.70
1:1	359.55	140.45
1:2	280.70	219.30
1:4	195.12	304.88
1:6	149.53	350.47
1:10	101.91	398.09

C

Molar ratio CA:PAN	Mass of CA [mg]	Mass of PAN [mg]
10:1	468.29	31.71
6:1	449.30	50.70
4:1	427.62	72.38
2:1	373.54	126.46
1:1	298.14	201.86
1:2	212.39	287.61
1:4	134.83	365.17
1:6	98.77	401.23
1:10	64.34	435.66

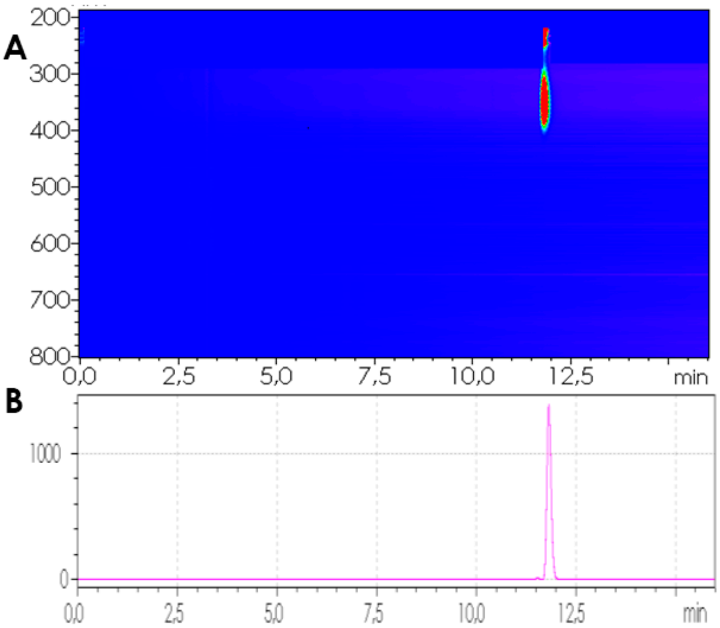

Figure S1. LC-DAD chromatogram of pure fluorophore separated from the reaction mixture (retention time: 11.8 min.) (**A**). LC chromatogram of the pure fluorophore calculated from a maximum plot of 190-800 nm (retention time: 11.8 min.) (**B**).

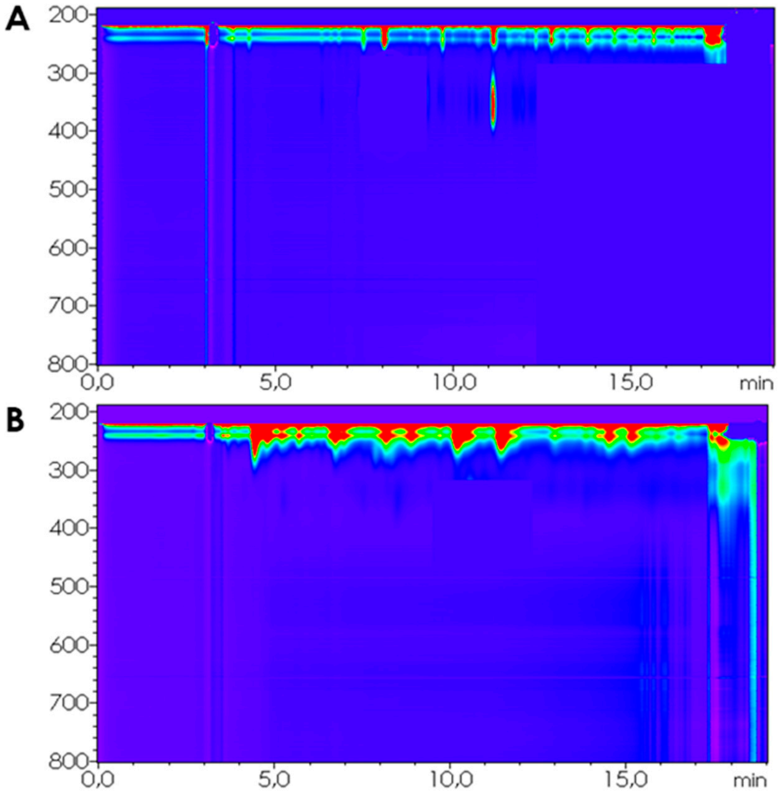

Figure S2. LC-DAD chromatogram of CA:3A1P reaction mixture (1:1 mol/mol. 160 °C. 180 min) (**A**). and LC-DAD chromatogram of CA:PAN reaction mixture (1:1 mol/mol. 160 °C. 180 min) (**B**).

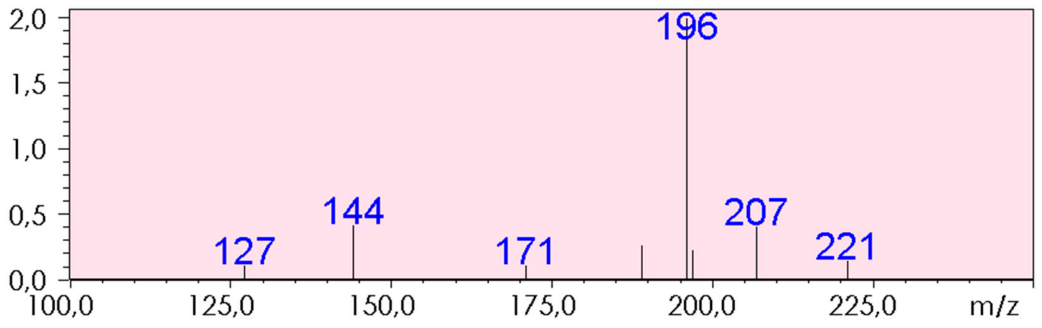

Figure S3. The mass spectrum of the fraction with a retention time of 11.8 minutes formed in a reaction with a molar ratio from CA:31AP 1:1.

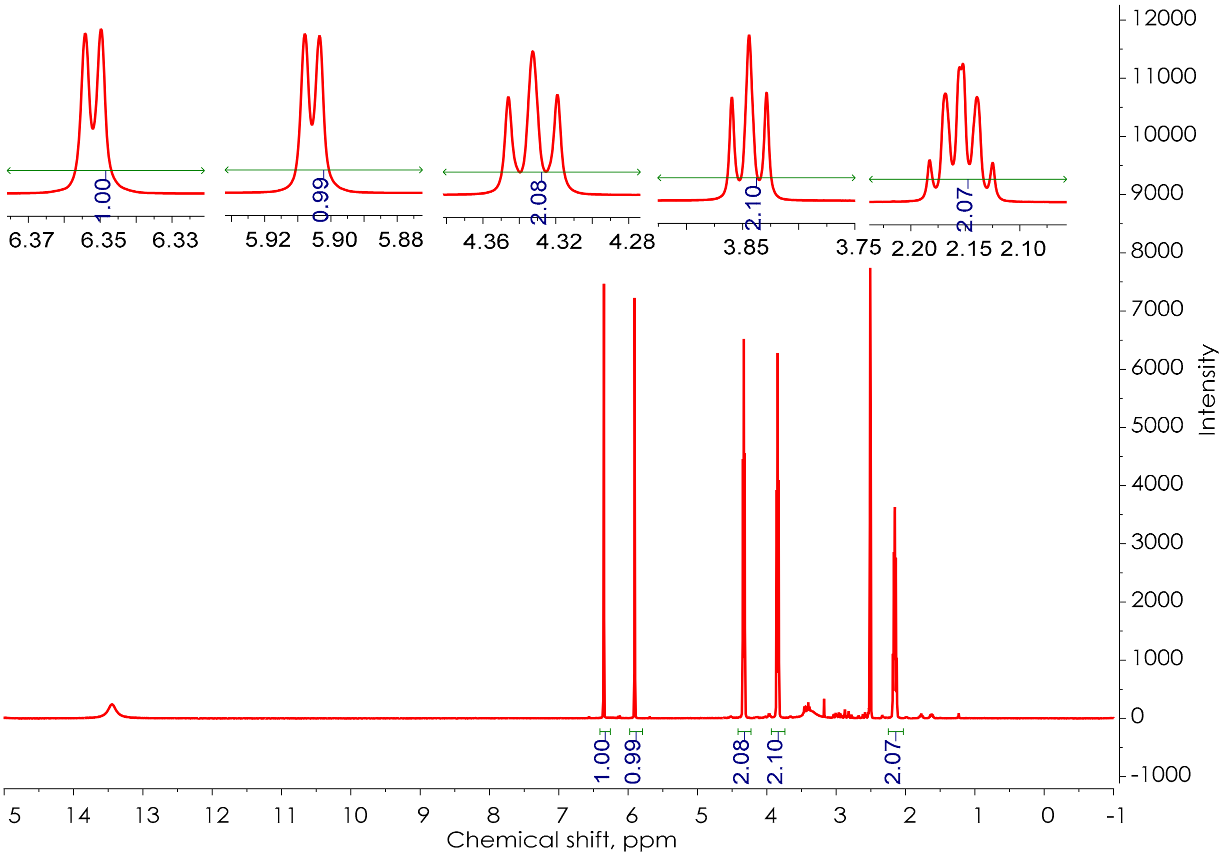

Figure S4. ^{1}H NMR of the fluorescent fraction.

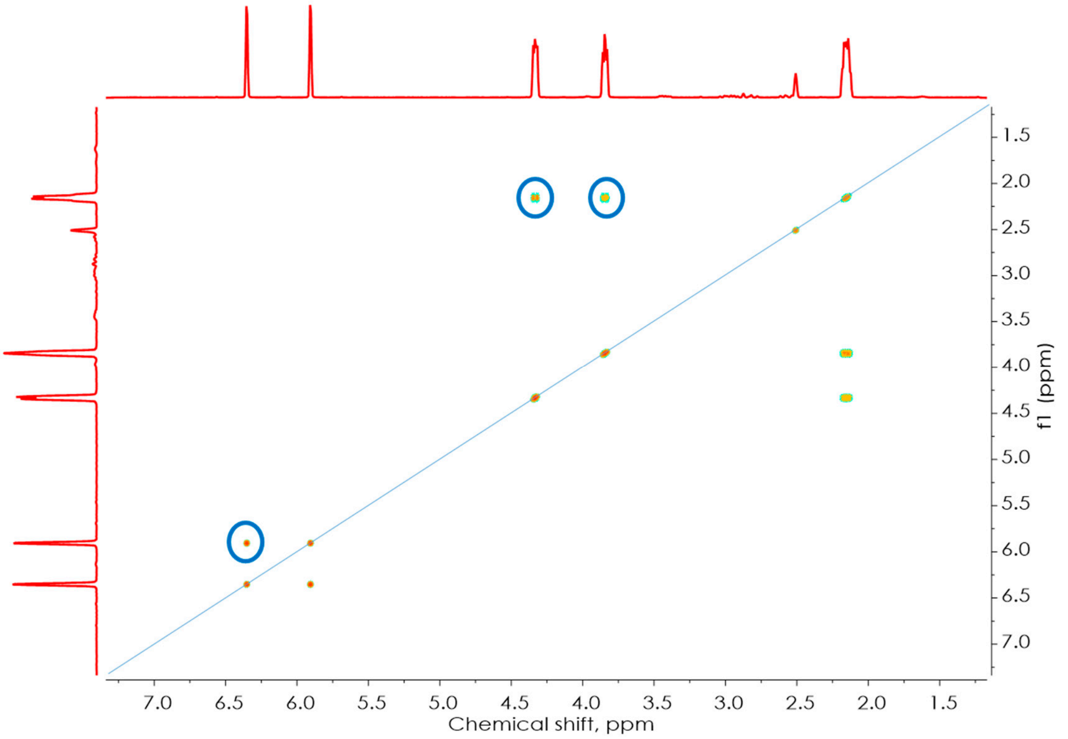

Figure S5. ^{1}H-^{1}H COSY NMR of the fluorescent fraction.

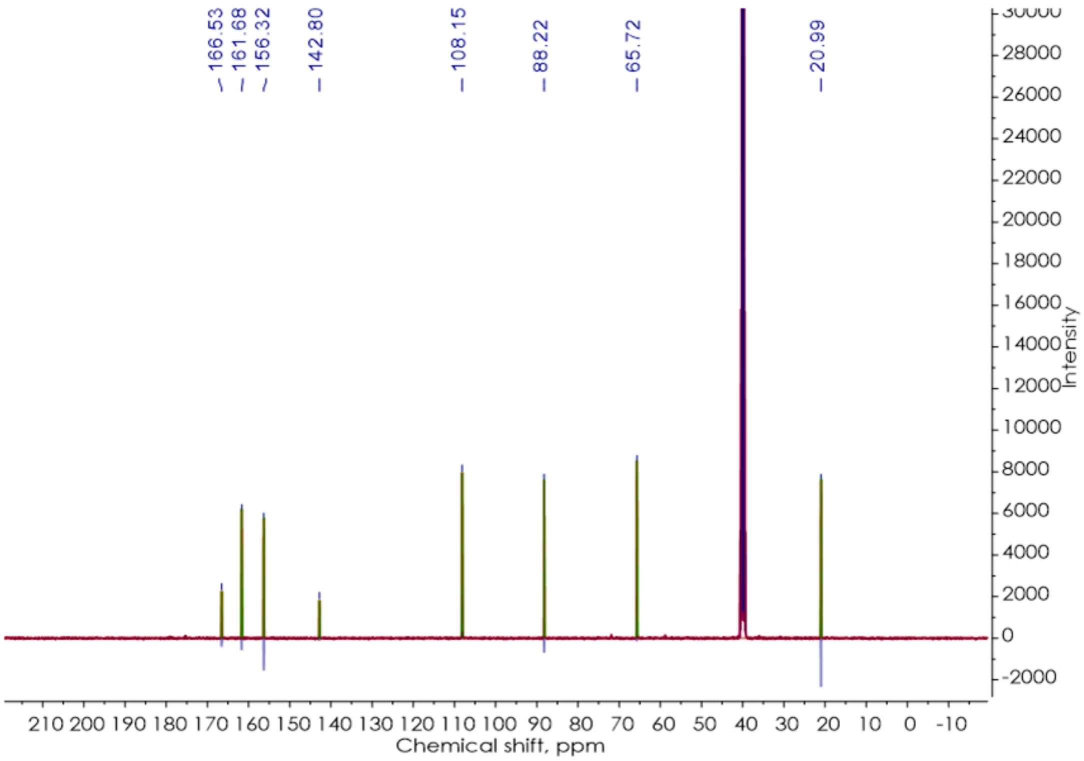

Figure S6. ^{13}C NMR of the fluorescent fraction.

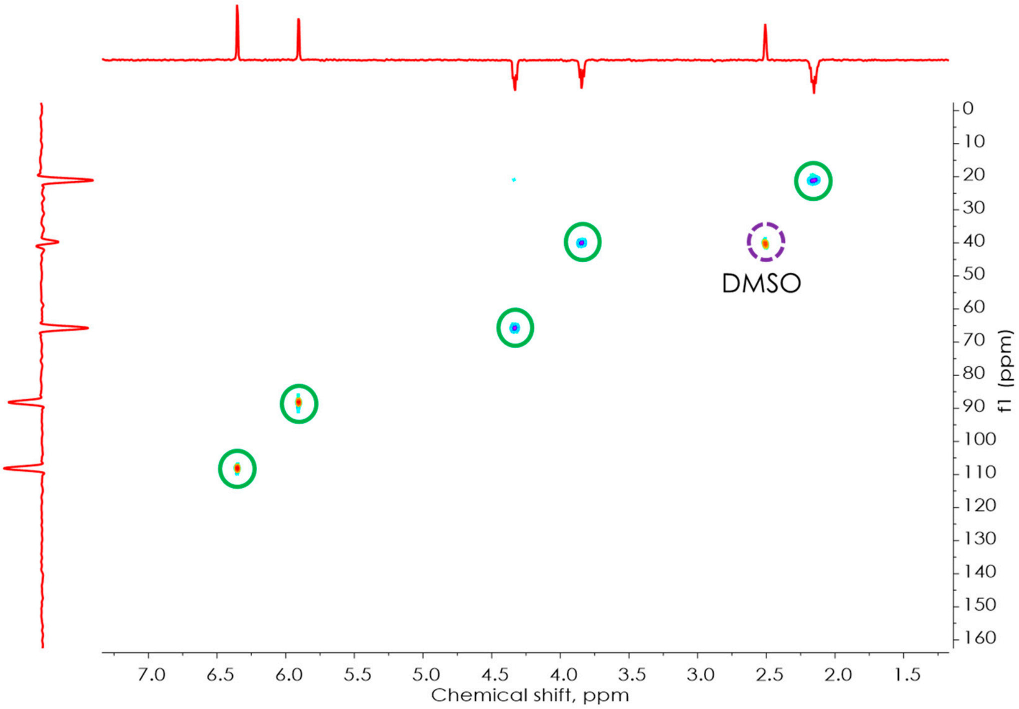

Figure S7. ^{1}H-^{13}C HSQC NMR of the fluorescent fraction.

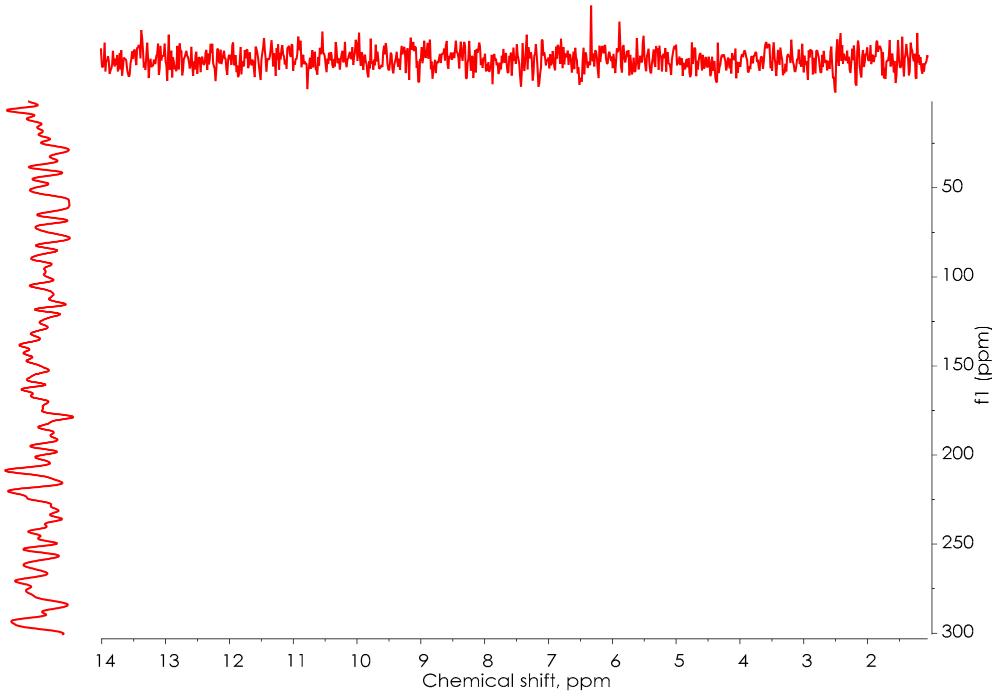

Figure S8. ^{1}H-^{15}N HSQC of the fluorescent fraction.

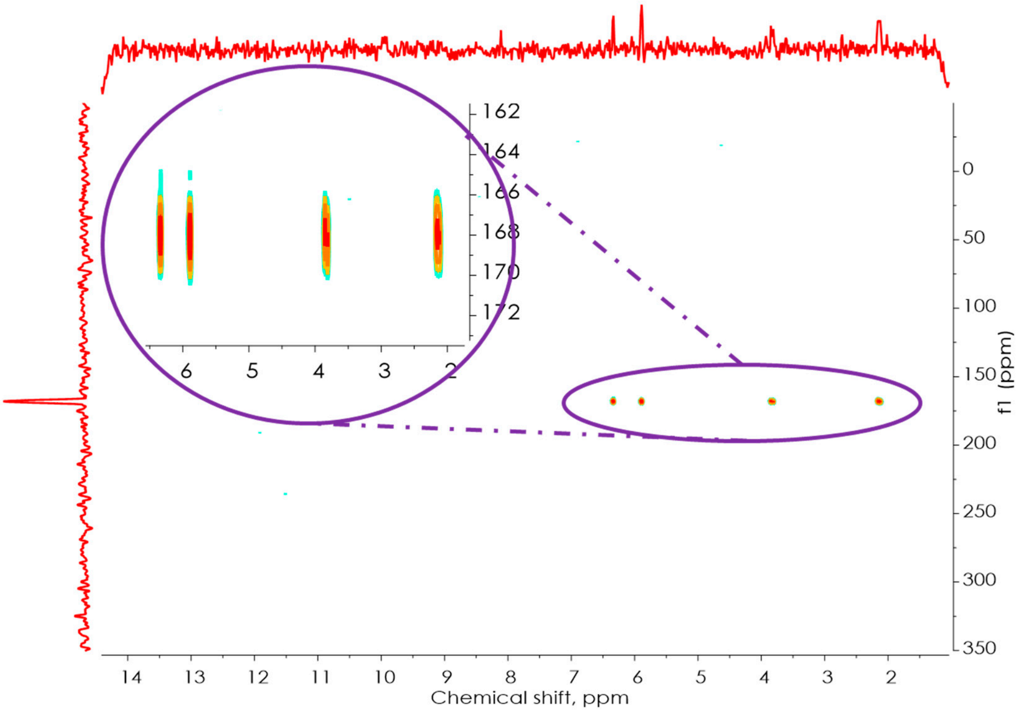

Figure S9. ^{1}H-^{15}N HMBC of the fluorescent fraction.

Table S2. Couplings between the atoms in ^{1}H-^{13}C HMBC NMR of the fluorescent fraction.

No.	Chemical shift (hydrogen) (ppm)	Chemical shift (carbon) (ppm)	Distance (number of bonds)
**1**	6.35	166.54	3
**2**	5.90	166.54	3
**3**	6.35	161.73	2
**4**	3.84	161.73	3
**5**	6.35	156.31	4
**6**	5.90	156.31	2
**7**	4.33	156.31	3
**8**	3.84	156.31	3
**9**	6.35	142.86	2
**10**	5.90	142.86	2
**11**	3.84	142.86	5
**12**	5.90	108.14	3
**13**	6.35	88.21	3
**14**	3.84	65.65	3
**15**	2.14	65.65	2
**16**	4.33	40.25	3
**17**	2.14	40.25	2
**18**	4.33	20.95	2
**19**	3.84	20.95	2

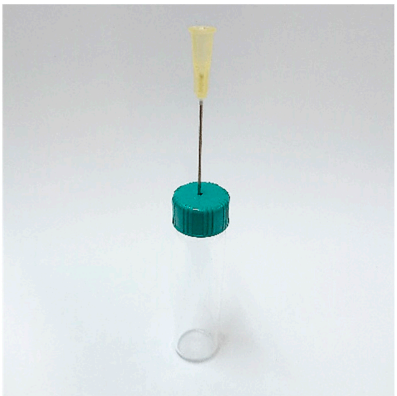

Figure S10. The type of vessel used in the process of panthenol derivatization.

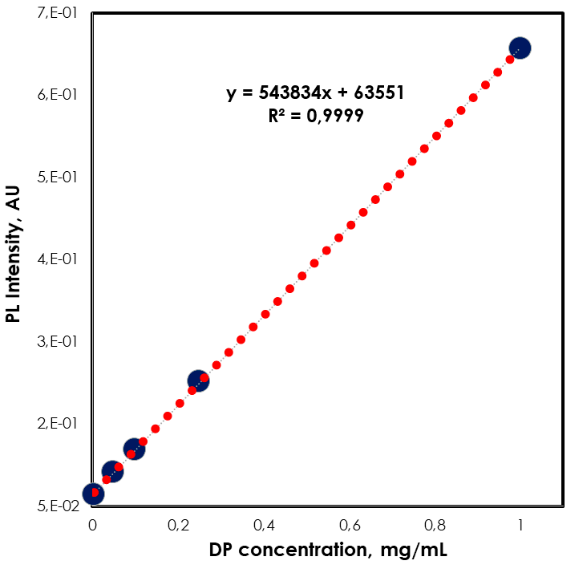

Figure S11. LC-MS calibration curve for the determination of panthenol in pharmaceutical formulations (calculated from the area under the curves).
